# Metformin Enhances 2-Aminoethyl Dihydrogen Phosphate-Induced Mitochondrial Dysfunction and Apoptosis in Melanoma Cells

**DOI:** 10.3390/ijms27125493

**Published:** 2026-06-18

**Authors:** Thalles Anthony Duarte de Oliveira, Gustavo Henrique Doná Rodrigues Almeida, Sergio Mestieri Chammas, Rosa Andrea Nogueira Laiso, Yasmim Emilly Moreira Sousa, Ícaro Gabriel Teles Pacheco de Matos, Valherya Silva Rodriguez, Beatriz Cristine Bittencourt Queiroz, Ariane Clemente Alves Oliveira, Sara de Lima, Laís Araujo Martins de Arruda, Daniel da Conceição Rabelo, Rose Eli Grassi Rici, Paulo Cézar de Freitas Mathias, Durvanei Augusto Maria

**Affiliations:** 1 Graduate Program in Anatomy of Domestic and Wild Animals, School of Veterinary Medicine and Animal Science, University of São Paulo, São Paulo 05508-270, Brazil; thalles.oliveira@pro.fecaf.com.br (T.A.D.d.O.); rosa.laiso@fundacaobutantan.org.br (R.A.N.L.); daniel.rabelo@butantan.gov.br (D.d.C.R.); roseeli@usp.br (R.E.G.R.); 2 Development and Innovation Laboratory, Butantan Institute, São Paulo 05503-900, Brazil; sm.chammas@alumni.usp.br (S.M.C.); yasmimmoreira@uni9.edu.br (Y.E.M.S.); i.matos.proppg@proppg.butantan.gov.br (Í.G.T.P.d.M.); svalherya@gmail.com (V.S.R.); biabittencourt0426@gmail.com (B.C.B.Q.); aric2802@gmail.com (A.C.A.O.); dsara2473@gmail.com (S.d.L.); laism1409@gmail.com (L.A.M.d.A.); 3 Graduate Program in Biological Sciences, State University of Maringá, Maringá 87020-900, Brazil; pmathias@uem.br; 4 Multidisciplinary Institute of Health, Federal University of Bahia, Vitória da Conquista 45029-094, Brazil

**Keywords:** melanoma, 2-AEH_2_P, metformin, tumor metabolism

## Abstract

Melanoma exhibits pronounced metabolic plasticity and mitochondrial dependency, contributing to therapeutic resistance and tumor progression. Targeting mitochondrial function therefore represents a promising anticancer strategy. 2-Aminoethyl dihydrogen phosphate (2-AEH_2_P), a bioactive phosphomonoester, has demonstrated antiproliferative potential, while metformin, a clinically established antidiabetic drug, acts as a mitochondrial complex I inhibitor and metabolic modulator. This study investigated the cytotoxic and mechanistic effects of 2-AEH_2_P and metformin hydrochloride, individually and in combination, in human (SK-MEL-28) and murine (B16-F10) melanoma models, using non-tumorigenic fibroblasts (FN1 and L929) as controls. Cell viability, proliferation dynamics, cell-cycle distribution, mitochondrial membrane potential (ΔΨm), and apoptosis-associated markers were evaluated by flow cytometry. 2-AEH_2_P reduced melanoma cell viability and proliferation while inducing G2/M accumulation, DNA fragmentation, mitochondrial depolarization, increased cytochrome c release, caspase-3 and caspase-8 activation, upregulation of p53 and Bad, and downregulation of Bcl-2. Metformin alone exerted moderate cytotoxic and pro-apoptotic effects. Notably, combined treatment markedly potentiated mitochondrial depolarization and intrinsic apoptotic signaling in melanoma cells, significantly lowering IC_50_ values and enhancing caspase activation and cytochrome c release. Bliss independence analysis demonstrated synergistic interaction in SK-MEL-28 and B16-F10 cells. Although interaction scores indicated synergy in one fibroblast model, absolute cytotoxicity remained lower than in melanoma cells. These findings demonstrate that metabolic co-targeting with metformin enhances mitochondrial dysfunction-associated apoptotic signaling in melanoma cells, supporting a drug repositioning strategy aimed at exploiting mitochondrial vulnerability in metabolically adaptable tumors.

## 1. Introduction

Cutaneous melanoma represents a biologically complex malignancy in which tumor progression, therapeutic resistance, and phenotypic plasticity are tightly intertwined with metabolic adaptability [1]. While oncogenic mutations such as BRAF and NRAS have historically shaped therapeutic strategies, increasing evidence indicates that melanoma survival is not solely mutation-driven but profoundly dependent on dynamic bioenergetic regulation [2,3]. Rather than conforming to a fixed metabolic phenotype, melanoma cells exhibit remarkable metabolic plasticity, enabling reconfiguration of energy production pathways in response to environmental stress and therapeutic pressure [2]. This adaptive flexibility has emerged as a critical determinant of disease aggressiveness and resistance.

In contrast to tumors that rely predominantly on aerobic glycolysis, melanoma frequently adopts hybrid metabolic states characterized by coordinated engagement of glycolysis and mitochondrial oxidative phosphorylation [4,5]. Subpopulations with enhanced mitochondrial respiration have been associated with increased invasive capacity, survival advantage, and resistance to targeted therapies [6]. These observations underscore the functional relevance of mitochondrial metabolism in melanoma biology. Mitochondrial dependency has therefore been proposed as a potential therapeutic vulnerability in melanoma [7].

Beyond ATP production, mitochondria function as integrative hubs regulating redox balance, reactive oxygen species (ROS) signaling, and apoptotic execution [8]. The intrinsic apoptotic pathway is critically dependent on mitochondrial outer membrane permeabilization [9]. Disruption of mitochondrial membrane potential (ΔΨm) facilitates the release of cytochrome c into the cytosol [10]. This event promotes apoptosome assembly and downstream activation of caspase cascades [10]. The susceptibility of tumor cells to mitochondrial apoptosis is governed by the balance between pro- and anti-apoptotic members of the Bcl-2 protein family [11]. Perturbation of mitochondrial homeostasis is therefore recognized as a mechanistically coherent strategy for inducing tumor-selective apoptosis [7,9,11].

Targeting tumor metabolism has gained increasing prominence as a complementary anticancer strategy [12]. Within this framework, drug repositioning offers an attractive avenue by leveraging compounds with established pharmacokinetic and safety profiles for new oncological applications [13]. Metformin, a biguanide extensively prescribed for the treatment of type 2 diabetes, has emerged as one of the most investigated repositioned agents in oncology [14]. Rather than acting as a conventional cytotoxic drug, metformin is recognized primarily as a modulator of cellular bioenergetics [14].

At the mechanistic level, metformin exerts its principal intracellular effects through functional inhibition of mitochondrial respiratory chain complex I [15]. This interference reduces ATP synthesis and induces energetic stress [15]. The resulting increase in the AMP/ATP ratio activates adaptive metabolic signaling pathways, including AMPK-dependent responses [16]. Experimental evidence indicates that metformin can suppress melanoma cell proliferation and modulate cell-cycle progression [17,18,19]. Studies further report that metformin exposure is associated with mitochondrial depolarization and enhanced activation of apoptosis-related pathways in melanoma models [20,21,22]. Increased cytochrome c release and caspase activation have been described following metformin-induced metabolic stress [17]. These findings suggest that melanoma cells exhibiting mitochondrial dependency may be particularly susceptible to bioenergetic disruption [23].

2-Aminoethyl dihydrogen phosphate (2-AEH_2_P), a bioactive phosphomonoester, represents an additional compound of interest within a mitochondrial-centered therapeutic framework [24]. Previous investigations have associated 2-AEH_2_P with antiproliferative effects and induction of apoptotic responses in tumor cells [25,26,27,28,29]. Observations of mitochondrial membrane depolarization following exposure to this compound suggest interference with mitochondrial integrity [24]. Modulation of apoptosis-related proteins, including members of the Bcl-2 family and effector caspases, has also been reported [26]. Although its precise molecular targets remain incompletely characterized, available evidence indicates that 2-AEH_2_P may perturb cellular homeostasis in a manner compatible with mitochondrial targeting.

Given the metabolic plasticity of melanoma and the central role of mitochondrial regulation in tumor survival, simultaneous interference with bioenergetic control and mitochondrial stability may amplify intrinsic apoptotic signaling [30]. Combining a metabolic disruptor such as metformin with a bioactive phosphomonoester capable of modulating mitochondrial integrity represents a mechanistically rational strategy. Such an approach aligns with contemporary paradigms emphasizing mitochondrial vulnerability and metabolic reprogramming as actionable targets in cancer therapy [24].

Based on these considerations, the study investigated the cellular effects of 2-AEH_2_P and metformin hydrochloride, individually and in combination, in human and murine melanoma models. Particular emphasis was placed on evaluating cytotoxicity, proliferative dynamics, cell-cycle modulation, mitochondrial membrane potential (ΔΨm), DNA fragmentation, and expression of apoptosis-associated markers. Pharmacological interactions were further characterized to determine whether combined metabolic and mitochondrial perturbation enhances tumor-selective responses.

## 2. Results

### 2.1. Evaluation of the Cytotoxic and Selective Effects of 2-Aminoethyl Dihydrogen Phosphate and Metformin Hydrochloride in Melanoma and Non-Tumorigenic Fibroblast Models

**Figure 1:**
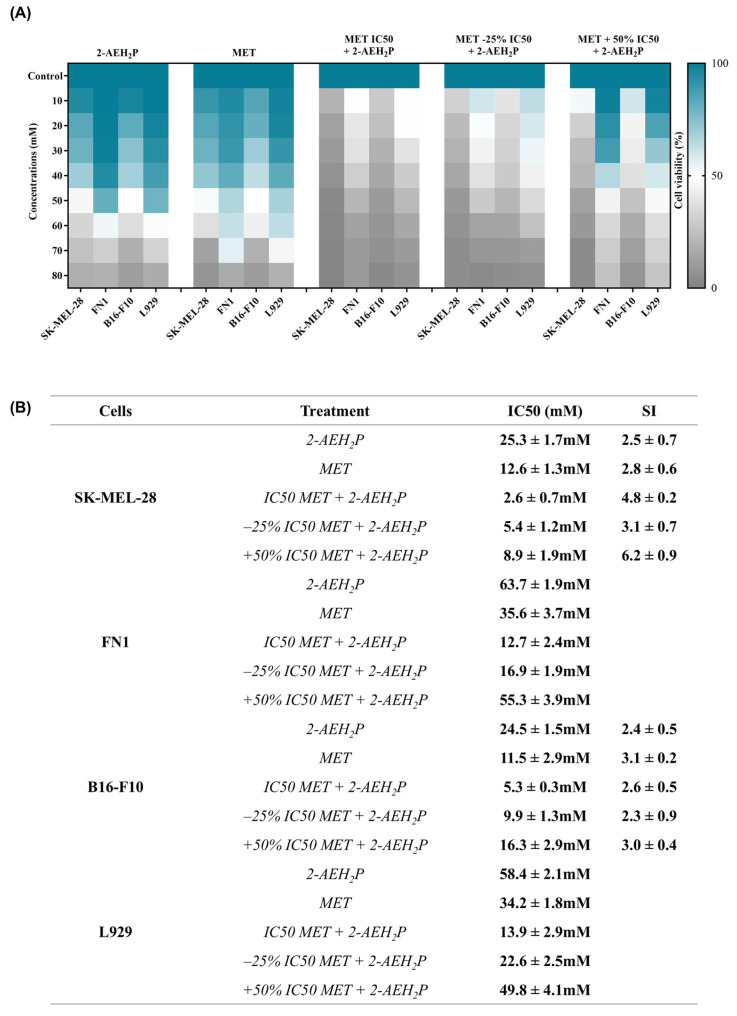
Cytotoxic profile and selectivity analysis of 2-AEH_2_P and metformin hydrochloride in melanoma and fibroblast models. (**A**) Heatmap representation of relative cell viability in SK-MEL-28 and B16-F10 melanoma cells, as well as FN1 and L929 non-tumorigenic fibroblasts, following 24 h exposure to escalating concentrations of 2-aminoethyl dihydrogen phosphate (2-AEH_2_P), metformin hydrochloride (MET), or their combination. The blue–white–gray gradient denotes the spectrum of viability responses across treatment conditions. Results correspond to the mean of three independent biological experiments conducted in technical triplicate (*n* = 3). (**B**) Half-maximal inhibitory concentration (IC_50_, mM) values and corresponding Selectivity Index (SI), calculated by comparing responses in melanoma cells to those observed in fibroblast controls. Data are presented as mean ± standard deviation. Statistical significance was determined by one-way ANOVA followed by Tukey’s post hoc multiple-comparison test, with a significance threshold set at *p* ≤ 0.05.

Treatment with 2-aminoethyl dihydrogen phosphate (2-AEH_2_P) alone produced marked cytotoxic effects in melanoma cells (Figure 1). The IC_50_ values were 25.3 ± 1.7 mM for SK-MEL-28 and 24.5 ± 1.5 mM for B16-F10 cells. In contrast, non-tumorigenic fibroblast controls exhibited substantially higher IC_50_ values, reaching 63.7 ± 1.9 mM in FN1 and 58.5 ± 2.1 mM in L929 cells, indicating preferential sensitivity of melanoma cells to 2-AEH_2_P. Metformin hydrochloride alone also reduced melanoma cell viability, with IC_50_ values of 12.6 ± 1.3 mM in SK-MEL-28 and 11.5 ± 2.9 mM in B16-F10 cells. Fibroblast controls were comparatively less sensitive, displaying IC_50_ values of 35.6 ± 3.7 mM in FN1 cells, while L929 cells exhibited minimal responsiveness under the tested conditions.

Notably, combined treatment with 2-AEH_2_P and metformin resulted in a pronounced enhancement of cytotoxic activity in melanoma models. In SK-MEL-28 cells, the IC_50_ values decreased to 2.6 ± 0.7 mM, 5.4 ± 1.2 mM, and 8.9 ± 1.9 mM under IC_50_-based, −25%, and +50% concentration conditions, respectively. Similarly, in B16-F10 melanoma cells, the combination reduced IC_50_ values to 5.3 ± 0.3 mM, 9.9 ± 1.3 mM, and 16.3 ± 2.9 mM across the same treatment schemes. In fibroblast controls, the combined treatment exhibited comparatively higher IC_50_ values. In FN1 cells, IC_50_ values were 12.7 ± 2.4 mM, 16.9 ± 1.9 mM, and 55.3 ± 3.9 mM, whereas in L929 cells the corresponding values were 13.9 ± 2.9 mM, 22.6 ± 2.5 mM, and 49.8 ± 4.1 mM. These findings indicate that the association of 2-AEH_2_P with metformin markedly enhances cytotoxicity in melanoma cells while maintaining comparatively reduced effects in non-tumorigenic fibroblasts, supporting a preferential cytotoxic profile.

### 2.2. Treatment-Induced Morphological Changes in SK-MEL-28 and B16-F10 Melanoma Cells Following Exposure to Metformin and Combined Treatment with 2-Aminoethyl Dihydrogen Phosphate

**Figure 2:**
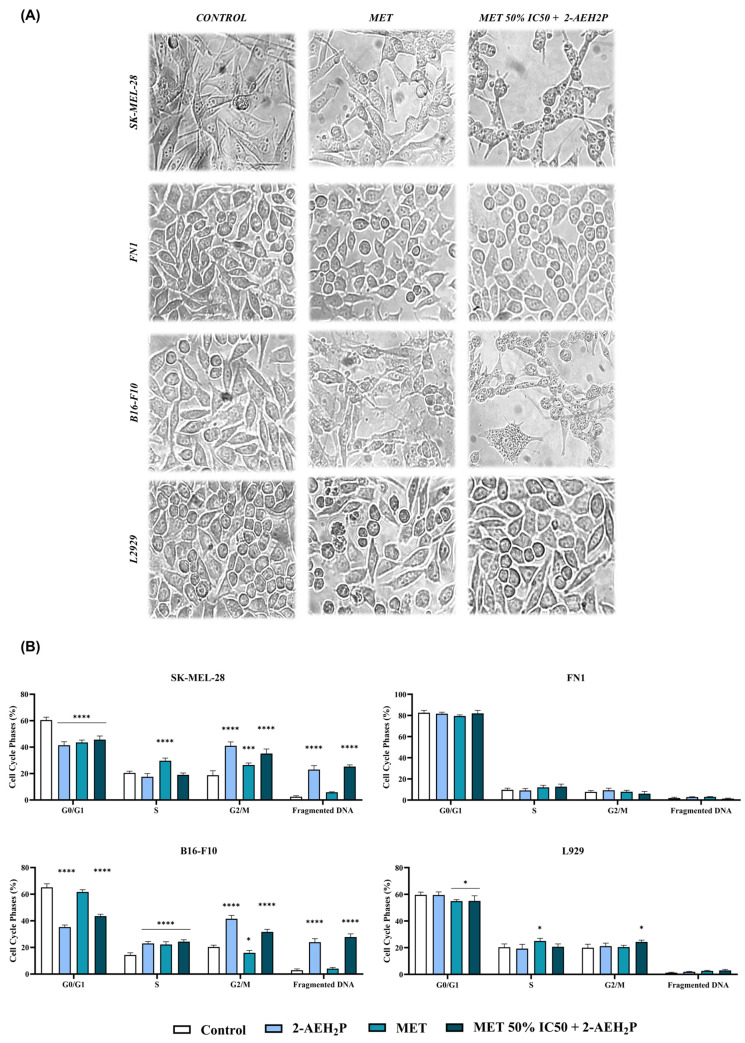
Morphological alterations and cell-cycle modulation induced by metformin and its combination with 2-AEH_2_P in melanoma and fibroblast cells. (**A**) Representative phase-contrast micrographs of SK-MEL-28 and B16-F10 melanoma cells, and FN1 and L929 fibroblasts, under control conditions, metformin treatment, and combined treatment (metformin 50% IC_50_ + 2-AEH_2_P). The combined treatment promotes pronounced morphological disruption in melanoma cells, including loss of adhesion, cell rounding, and formation of cellular aggregates, while fibroblasts remain comparatively preserved. Scale bar: 20 µm. (**B**) Quantitative analysis of cell-cycle distribution under identical experimental conditions, showing the proportion of cells in G_0_/G_1_, S, G_2_/M phases, and fragmented DNA. Data are presented as mean ± standard deviation from three independent biological experiments performed in technical triplicate (*n* = 3). Statistical analysis was performed using one-way ANOVA followed by Tukey’s multiple-comparison post hoc test. Differences relative to untreated controls were considered statistically significant at * *p* < 0.05, *** *p* < 0.001, and **** *p* < 0.0001.

Marked structural alterations were observed in melanoma cells following treatment with metformin alone or in combination with 2-AEH_2_P (Figure 2A). In SK-MEL-28 cultures, control cells displayed a typical adherent, elongated morphology, whereas metformin-treated cells exhibited mild morphological changes, including partial cell rounding and reduced spreading. In contrast, combined treatment (metformin 50% IC_50_ + 2-AEH_2_P) resulted in pronounced structural disruption, characterized by loss of adhesion, extensive cell rounding, and the presence of non-adherent cellular aggregates, consistent with cytotoxic morphology and features commonly associated with apoptotic progression. A similar pattern was observed in B16-F10 melanoma cells, in which the combined treatment induced marked disorganization of the monolayer, increased cell detachment, and the appearance of clustered, rounded cells. In both melanoma models, these alterations were substantially more pronounced under combined exposure compared to metformin alone, consistent with the enhanced cell-cycle disruption and mitochondrial effects observed under 2-AEH_2_P-containing conditions. In contrast, fibroblast cell lines (FN1 and L929) demonstrated greater morphological preservation across all conditions. Control and metformin-treated fibroblasts maintained their characteristic elongated morphology and adherence, while combined treatment induced only modest changes, such as slight reductions in cell density, without evidence of extensive structural collapse or widespread detachment.

### 2.3. Modulation of Cell-Cycle Distribution in Melanoma and Fibroblast Cells Induced by 2-Aminoethyl Dihydrogen Phosphate and Metformin

Exposure to 2-aminoethyl dihydrogen phosphate (2-AEH_2_P) significantly altered cell-cycle distribution in melanoma cells (Figure 2). In SK-MEL-28 cells, treatment reduced the G0/G1 population by 19.1 ± 2.9% and increased the G2/M fraction by 22.2 ± 2.7%, indicating cell-cycle progression disturbance and accumulation in mitotic phases. Additionally, the proportion of cells with fragmented DNA increased by 20.5 ± 3.4%, supporting the induction of apoptosis-associated DNA damage. In contrast, FN1 fibroblast cells exhibited only minimal cell-cycle alterations under the same conditions. Similarly, in B16-F10 melanoma cells, 2-AEH_2_P markedly reduced the G0/G1 phase (28.2 ± 0.4%) while significantly increasing the S and G2/M fractions, consistent with proliferative disruption and cell-cycle arrest. In L929 fibroblasts, however, cell-cycle distribution remained largely unchanged, with only a slight increase in fragmented DNA (0.6 ± 0.1%), indicating limited impact on non-tumorigenic cells.

Metformin hydrochloride alone also modulated cell-cycle dynamics in melanoma models. In SK-MEL-28 cells, treatment reduced the G0/G1 phase by 17.0 ± 0.4% and increased the G2/M fraction by 15.0 ± 0.4%, suggesting interference with cell-cycle progression. In B16-F10 cells, isolated metformin exposure led to a significant reduction in the G0/G1 phase (13.2 ± 0.8%). In contrast, fibroblast controls displayed minimal or no significant alterations in cell-cycle phase distribution following metformin treatment.

The combined treatment with 2-AEH_2_P and metformin amplified cell-cycle disruption in melanoma cells. In SK-MEL-28 cells, the association induced a marked redistribution of cell populations, with accumulation in the G2/M phase and increased DNA fragmentation relative to single-agent treatments. In B16-F10 melanoma cells, the combined exposure intensified the reduction in G0/G1 (21.7 ± 0.4%) and significantly increased the S (7.8 ± 0.7%) and G2/M (4.3 ± 0.6%) phases, indicating enhanced cell-cycle perturbation. In fibroblast controls, the combined treatment produced comparatively modest effects. FN1 cells exhibited mild reductions in the G0/G1 phase under both isolated and combined conditions, while L929 cells showed no significant alterations in cell-cycle distribution.

### 2.4. Modulation of Proliferative Activity in Melanoma and Fibroblast Cells Induced by 2-Aminoethyl Dihydrogen Phosphate and Metformin

**Figure 3:**
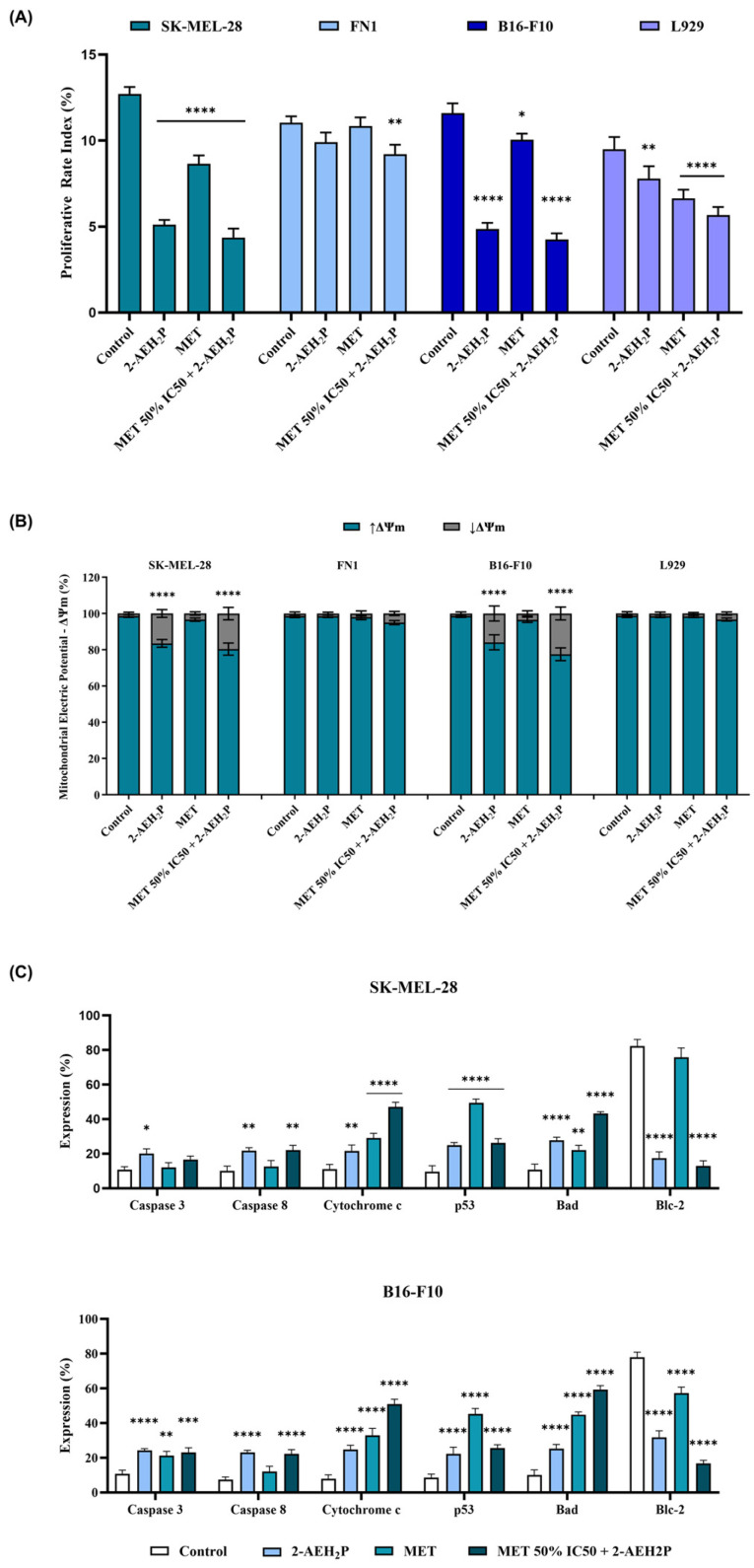
Impact of 2-AEH_2_P and metformin hydrochloride on proliferative capacity, mitochondrial membrane potential, and apoptosis-associated markers. (**A**) Proliferation analysis of SK-MEL-28 and B16-F10 melanoma cells, as well as FN1 and L929 fibroblast controls, following 24 h exposure to 2-aminoethyl dihydrogen phosphate (2-AEH_2_P), metformin hydrochloride, or their combined treatment (metformin at 50% of its IC_50_ plus 2-AEH_2_P). The proliferation index reflects treatment-dependent modulation of cell division across different cell types. (**B**) Assessment of mitochondrial membrane potential (ΔΨm), demonstrating variations in mitochondrial polarization status after treatment and indicating differential susceptibility between melanoma and fibroblast models. (**C**) Flow-cytometric quantification of apoptosis-related proteins, including caspase-3, caspase-8, cytochrome c, p53, Bad, and Bcl-2, illustrating treatment-induced modulation of intrinsic apoptotic signaling in melanoma cells. Results are presented as relative changes in mean fluorescence intensity (MFI) compared to untreated controls. Data represent mean ± standard deviation from three independent biological experiments performed in technical triplicate (*n* = 3). Statistical comparisons were conducted using one-way ANOVA followed by Tukey’s multiple-comparison post hoc test. Differences relative to untreated controls were considered significant at * *p* < 0.05, ** *p* < 0.01, *** *p* < 0.001, and **** *p* < 0.0001.

Treatment with 2-aminoethyl dihydrogen phosphate (2-AEH_2_P) alone significantly reduced the proliferative index of melanoma cells (Figure 3A). In SK-MEL-28 cells, the proliferative index decreased to 4.9 ± 0.9, and in B16-F10 cells to 5.1 ± 1.5. In contrast, fibroblast controls maintained higher proliferative indices, with values of 9.5 ± 0.9 in FN1 and 7.2 ± 1.5 in L929 cells, indicating preferential antiproliferative activity in melanoma models. Metformin hydrochloride alone exerted only modest effects on cellular proliferation. The proliferative indices were 8.3 ± 0.9 in SK-MEL-28 and 9.8 ± 1.0 in B16-F10 melanoma cells. Similarly, fibroblast controls exhibited limited changes, with indices of 10.4 ± 1.2 in FN1 and 8.1 ± 1.4 in L929 cells, suggesting that metformin as a single agent had relatively mild impact on proliferative dynamics under the tested conditions. The combined treatment with 2-AEH_2_P and metformin markedly enhanced antiproliferative effects in melanoma cells. In SK-MEL-28 cells, the proliferative index decreased to 4.7 ± 1.1, while in B16-F10 cells it was further reduced to 3.9 ± 1.2, reflecting a pronounced suppression of cell division. In contrast, fibroblast controls showed no significant reduction in proliferative activity following combination treatment, maintaining indices of approximately 10 in FN1 and 8.5 ± 1.2 in L929 cells.

### 2.5. Modulation of Mitochondrial Membrane Potential (ΔΨm) in Melanoma and Fibroblast Cells Induced by 2-Aminoethyl Dihydrogen Phosphate and Metformin

After 24 h of exposure to 2-AEH_2_P alone, SK-MEL-28 and B16-F10 melanoma cells exhibited a reduction in mitochondrial membrane potential (ΔΨm) of 15.1 ± 0.5% and 14.6 ± 1.5%, respectively (Figure 3B). In FN1 and L929 fibroblasts, no significant changes were detected, with minimal reductions of 0.2 ± 0.1% and 0.3 ± 0.2%, respectively. Metformin hydrochloride alone induced modest mitochondrial depolarization in melanoma cells, reducing ΔΨm by 2.1 ± 0.2% in SK-MEL-28 and 2.2 ± 0.2% in B16-F10 cells. In fibroblast controls, reductions remained minimal (0.6 ± 0.3% in FN1 and 0.4 ± 0.1% in L929). Combined treatment markedly potentiated mitochondrial dysfunction in melanoma cells. In SK-MEL-28 cells, ΔΨm reduction reached 27.1 ± 1.4%, while in B16-F10 cells the reduction was 23.1 ± 1.9%. In contrast, fibroblast cells exhibited substantially lower responses under combined exposure. FN1 cells showed reductions of 3.1 ± 0.1%, and L929 cells displayed a reduction of 2.8 ± 0.2%, values that remained markedly inferior to those observed in melanoma models.

### 2.6. Induction of Mitochondria-Mediated Intrinsic Apoptosis by 2-Aminoethyl Dihydrogen Phosphate and Metformin in Melanoma Cells

Treatment with 2-aminoethyl dihydrogen phosphate (2-AEH_2_P) alone significantly activated apoptosis-associated signaling in melanoma cells (Figure 3C). In SK-MEL-28 cells, caspase-3 (9.2 ± 1.0%) and caspase-8 (11.8 ± 0.5%) expression increased, accompanied by enhanced cytochrome c release (10.4 ± 1.4%). Pro-apoptotic regulators p53 (15.3 ± 0.4%) and Bad (17.0 ± 0.6%) were upregulated, whereas the anti-apoptotic protein Bcl-2 was markedly reduced (64.9 ± 1.9%), indicating activation of the intrinsic apoptotic pathway. A comparable pattern was observed in B16-F10 cells, with increased caspase-3 (10.8 ± 0.3%), caspase-8 (7.4 ± 0.4%), and cytochrome c release (7.8 ± 0.9%), alongside upregulation of p53 (8.5 ± 1.9%) and Bad (10.2 ± 1.1%), and a substantial reduction in Bcl-2 levels (78.0 ± 1.8%).

Metformin hydrochloride alone exerted comparatively moderate pro-apoptotic modulation. In SK-MEL-28 cells, caspase-3 (1.2 ± 1.0%) and caspase-8 (2.5 ± 1.5%) showed limited increases, while cytochrome c release (17.9 ± 0.9%) and p53 expression (39.8 ± 0.8%) were more prominently elevated. In B16-F10 cells, metformin alone increased caspase-3 (10.4 ± 1.2%) and caspase-8 (4.7 ± 1.5%), accompanied by cytochrome c release (25.0 ± 2.1%) and marked p53 upregulation (36.7 ± 1.8%). Although Bcl-2 levels were reduced (20.6 ± 0.6%), the overall apoptotic profile remained partial compared to 2-AEH_2_P treatment.

Combined treatment with 2-AEH_2_P and metformin enhanced key mitochondrial apoptotic events in both melanoma models. In SK-MEL-28 cells, the association substantially increased cytochrome c release (35.8 ± 1.2%) and Bad expression (32.5 ± 0.2%), while strongly reducing Bcl-2 levels (78.5 ± 2.8%). Caspase-8 activation (12.0 ± 1.1%) was maintained at levels comparable to or slightly above single treatments, whereas caspase-3 (6.0 ± 0.8%) did not exceed the levels induced by 2-AEH_2_P alone. Although p53 expression was more pronounced under isolated metformin exposure, the combined regimen sustained a robust pro-apoptotic shift characterized by mitochondrial depolarization, cytochrome c mobilization, and suppression of anti-apoptotic signaling. Similarly, in B16-F10 cells, the combination further increased caspase-3 (12.3 ± 1.4%) and caspase-8 (14.8 ± 1.4%) relative to single treatments. Cytochrome c release was markedly elevated (43.2 ± 1.3%), Bad expression increased (49.2 ± 0.8%), and Bcl-2 was significantly reduced (61.2 ± 1.7%), reinforcing the amplification of intrinsic apoptotic signaling under combined metabolic and mitochondrial perturbation.

### 2.7. Pharmacological Interaction Between 2-Aminoethyl Dihydrogen Phosphate and Metformin in Melanoma Cells

**Figure 4:**
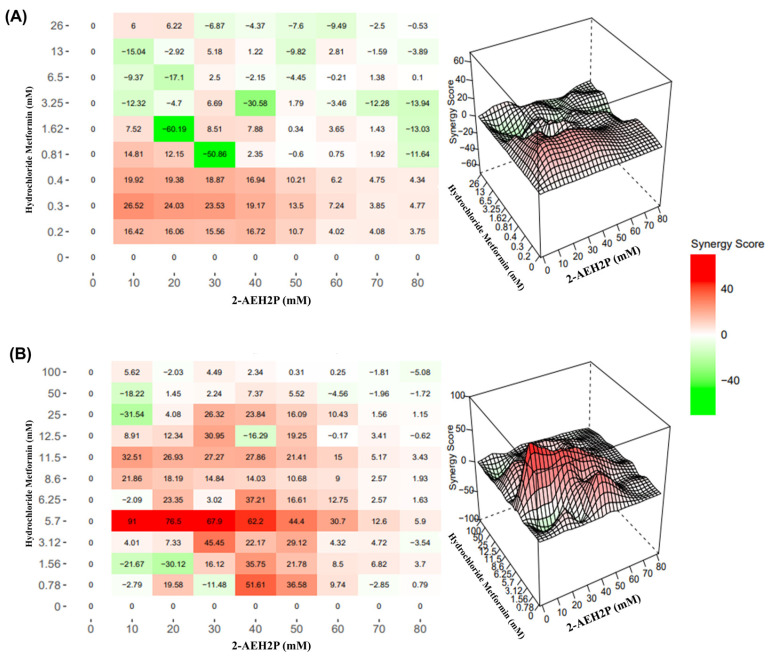
Pharmacological interaction analysis of 2-AEH_2_P and metformin hydrochloride in melanoma models. Bliss independence interaction matrices were constructed for SK-MEL-28 cells to evaluate how varying concentration pairings of 2-aminoethyl dihydrogen phosphate (2-AEH_2_P) and metformin hydrochloride affect cytotoxic outcomes across the experimental dose grid (**A**). The corresponding interaction landscape generated for B16-F10 cells (**B**) depicts the spatial distribution of interaction scores, distinguishing additive regions from concentration windows exhibiting synergistic amplification. The response patterns indicate that cooperative effects are dose-dependent rather than uniformly distributed across the matrix. These three-dimensional interaction surfaces support the interpretation that simultaneous induction of mitochondrial destabilization and bioenergetic interference enhances vulnerability of melanoma cells under combined treatment conditions.

Drug interaction analysis using the Bliss independence model (Figure 4) demonstrated a synergistic effect for the combination of 2-aminoethyl dihydrogen phosphate (2-AEH_2_P) and metformin hydrochloride in melanoma cells. In SK-MEL-28 cells, the combination yielded a synergy score of 18.94 ± 2.5, indicating strong synergistic interaction. Similarly, in B16-F10 melanoma cells, the mean Bliss score was 12.48 ± 1.6, also consistent with a synergistic effect. In non-tumorigenic fibroblast controls, interaction profiles differed between cell lines. In FN1 fibroblasts, the combination produced a Bliss score of 16.89 ± 2.1, suggesting synergistic interaction under the tested conditions. In contrast, L929 fibroblasts exhibited a mean score of 0.42 ± 0.9, indicating an overall additive interaction. According to established Bliss model thresholds (synergy > +10; additive between −10 and +10; antagonism < −10), these findings demonstrate that the association of 2-AEH_2_P and metformin produces a robust synergistic cytotoxic interaction in melanoma cells, while exerting additive or variable effects in fibroblast models.

## 3. Discussion

Metabolic adaptability represents a central determinant of melanoma aggressiveness and therapeutic resistance, positioning mitochondrial regulation as a strategic vulnerability rather than a secondary consequence of oncogenic signaling [31]. In this context, therapeutic strategies capable of simultaneously perturbing mitochondrial integrity and cellular bioenergetics have emerged as a rational approach to overcome tumor metabolic plasticity [32]. Based on this, drug repositioning has gained relevance not merely as a cost-reduction strategy, but as a mechanistically informed avenue to exploit tumor-specific metabolic dependencies [13].

Metformin represents one of the most extensively investigated repositioned drugs in oncology, largely due to its well-characterized safety profile and capacity to interfere with mitochondrial metabolism [33]. At the cellular level, metformin inhibits mitochondrial respiratory chain complex I, leading to decreased ATP production and increased energetic stress [15]. This metabolic perturbation has been associated with suppression of tumor cell proliferation, modulation of cell-cycle dynamics, and induction of apoptosis-related signaling in several cancer models, including melanoma [34]. Previous experimental studies have demonstrated that metformin can inhibit melanoma growth and trigger apoptosis-associated mechanisms, reinforcing the concept that bioenergetic disruption can sensitize tumor cells to mitochondrial dysfunction [17,18,35].

Our findings support the concept that mitochondrial destabilization constitutes a key mechanism underlying the cytotoxic effects of 2-AEH_2_P [24]. Exposure to this phosphomonoester induced mitochondrial membrane depolarization, cytochrome c mobilization, Bad upregulation, and pronounced reduction in the anti-apoptotic protein Bcl-2 [24]. These alterations are characteristic of mitochondrial outer membrane permeabilization, a pivotal event in the intrinsic apoptotic pathway [36]. The concomitant activation of caspase-3 and caspase-8 further supports the interpretation that mitochondrial permeabilization leads to downstream apoptotic signaling rather than nonspecific cellular toxicity [37]. These observations are consistent with previous work from our laboratory demonstrating that 2-AEH_2_P modulates mitochondrial stability and Bcl-2 family proteins while promoting apoptosis in melanoma and other tumor models [24].

In contrast to the direct mitochondrial destabilization induced by 2-AEH_2_P, metformin alone produced a distinct yet complementary cellular response. Although mitochondrial depolarization was relatively modest, metformin treatment markedly increased p53 expression and cytochrome c release, indicating induction of bioenergetic stress and partial activation of apoptotic pathways [38]. This pattern suggests that metformin does not necessarily execute apoptosis independently under these experimental conditions but instead primes tumor cells by shifting the intracellular energetic balance toward a pro-apoptotic state. Such metabolic priming may reduce the threshold required for mitochondrial permeabilization, thereby increasing cellular susceptibility to mitochondrial-targeted compounds [39].

The combined exposure of melanoma cells to 2-AEH_2_P and metformin markedly intensified mitochondrial dysfunction and apoptotic signaling. The association produced a greater reduction in mitochondrial membrane potential compared with either compound alone, accompanied by substantial cytochrome c mobilization and sustained suppression of Bcl-2. These events collectively support a model in which metabolic stress imposed by metformin enhances the pro-apoptotic effects of mitochondrial destabilization induced by 2-AEH_2_P [40]. Even in situations where caspase-3 activation did not exceed that observed with the phosphomonoester alone, upstream mitochondrial events were clearly amplified, indicating reinforcement of apoptotic commitment at the organelle level [41].

Drug interaction analysis further corroborated the existence of a functional synergy between metabolic stress and mitochondrial disruption. Bliss independence modeling demonstrated synergy scores exceeding +10 in both melanoma cell lines, indicating that the combined effect of the two compounds surpassed the theoretical additive expectation [42]. Mechanistically, this cooperative interaction can be interpreted as the simultaneous collapse of mitochondrial integrity and cellular energy homeostasis, thereby compressing the metabolic buffering capacity required for tumor cell survival [43]. The resulting energetic imbalance likely accelerates apoptotic execution by facilitating mitochondrial outer membrane permeabilization and apoptosome activation [43].

Although cooperative responses were detected in one fibroblast model, the magnitude of mitochondrial depolarization, apoptosis-associated signaling, and IC_50_ reduction remained substantially greater in melanoma cells. This differential sensitivity may reflect the increased mitochondrial dependency commonly observed in melanoma, where oxidative phosphorylation contributes to tumor progression and resistance to targeted therapies [44]. Consequently, tumor cells relying heavily on mitochondrial bioenergetics may be disproportionately susceptible to strategies combining metabolic interference with mitochondrial destabilization [45]. Although these findings were obtained using in vitro experimental models, the data provide mechanistic insight into how coordinated metabolic stress and mitochondrial destabilization can enhance intrinsic apoptotic signaling in melanoma cells. Further studies using in vivo systems will be necessary to determine whether this metabolic–mitochondrial interaction translates into therapeutic efficacy in more complex biological contexts.

## 4. Materials and Methods

**Figure 5:**
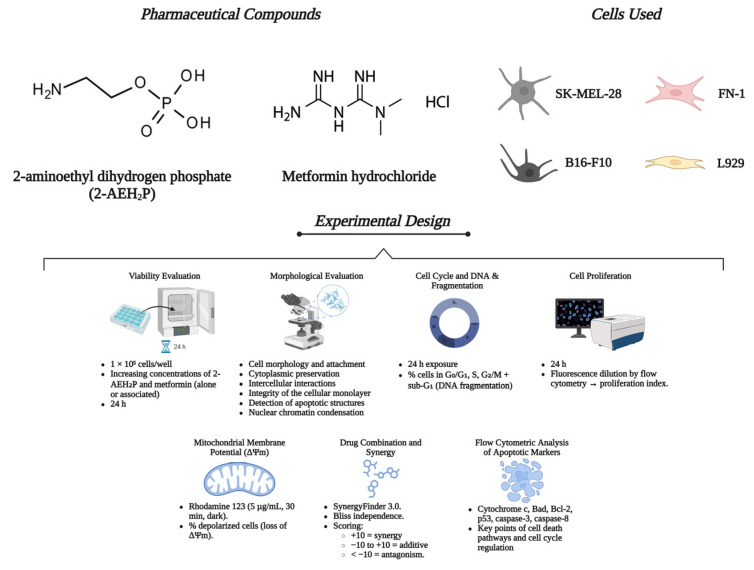
Experimental workflow outlining treatment conditions, cellular models, and analytical endpoints. Human and murine melanoma cell lines (SK-MEL-28 and B16-F10) together with non-tumorigenic fibroblast controls (FN1 and L929) were treated for 24 h with graded concentrations of 2-aminoethyl dihydrogen phosphate (2-AEH_2_P) and metformin hydrochloride, administered either individually or in combination. Following exposure, multiple cellular responses were examined, including viability, morphological alterations, proliferative behavior, cell-cycle distribution, DNA content, and mitochondrial membrane potential (ΔΨm). Apoptosis-associated markers were quantified by flow cytometry. Drug interaction patterns were evaluated using the Bliss independence reference model. Created in BioRender. Almeida, G. H. D. (2026) https://BioRender.com/8kc2han. (accessed on 1 March 2026).

This study evaluated the biological effects arising from the combination of 2-aminoethyl dihydrogen phosphate (2-AEH_2_P) and metformin hydrochloride in human and murine melanoma cell models. Multiple complementary assays were performed to characterize cytotoxic responses, modulation of proliferative capacity, perturbations in cell-cycle progression, and activation of apoptosis-associated pathways. Non-tumorigenic fibroblast cell lines were incorporated as comparative controls to examine the selectivity of the treatments. All experimental procedures were conducted under controlled culture conditions, with each assay performed in triplicate to ensure reproducibility. A schematic representation of the experimental design is shown in Figure 5.

### 4.1. Preparation of 2-Aminoethyl Dihydrogen Phosphate and Metformin Hydrochloride and Experimental Cell Culture Conditions

2-Aminoethyl dihydrogen phosphate (2-AEH_2_P) employed in this study was provided by PhosphoPure^®^ (São Paulo, Brazil) with certified analytical grade purity exceeding 99%, accompanied by the corresponding quality control documentation. For experimental use, the compound was solubilized in sterile ultrapure water to generate a 100 mM stock solution, followed by aliquoting under aseptic conditions and storage at 4 °C until application. Metformin hydrochloride was obtained from Sigma-Aldrich (St. Louis, MO, USA) and prepared immediately prior to use by dissolution in sterile phosphate-buffered saline (PBS, pH 7.4), yielding a stock solution of appropriate concentration. Complete solubilization was ensured by gentle vortexing, and the solution was sterilized through a 0.22 µm membrane filter. Human melanoma cells (SK-MEL-28) and murine melanoma cells (B16-F10) were utilized as tumor models. To evaluate treatment selectivity, non-tumorigenic fibroblast cell lines of human (FN1) and murine origin (L929) were included as reference controls. These fibroblast models were selected due to their preserved proliferative behavior, dynamic membrane remodeling, and metabolic adaptability, thereby enabling discrimination between tumor-specific responses and generalized cytotoxic effects. All cell lines were cultured in RPMI-1640 medium supplemented with L-glutamine, HEPES buffer, sodium bicarbonate, a standard penicillin/streptomycin antibiotic mixture, and fetal bovine serum. Cultures were maintained at 37 °C in a humidified incubator under 5% CO_2_. Cellular viability and membrane integrity were routinely monitored using the Trypan Blue exclusion method.

### 4.2. Assessment of Cell Viability

For viability measurements, cells were plated in 6-well culture plates at a density of 1 × 10^5^ cells per well and maintained for 24 h to ensure proper adhesion and recovery. Following this stabilization period, cultures were treated with graded concentrations of 2-aminoethyl dihydrogen phosphate (2-AEH_2_P; 1–100 mM) and metformin hydrochloride (0.1–20 mM), administered either as single agents or in combined regimens. After 24 h of exposure, cellular metabolic activity was quantified using the MTT reduction assay. The MTT reagent was prepared immediately before use in phosphate-buffered saline (PBS, pH 7.4) at a concentration of 5 mg/mL. At the end of the treatment period, conditioned medium was carefully aspirated, and cells were incubated with the MTT solution for 3 h at 37 °C under a humidified 5% CO_2_ atmosphere. The resulting formazan precipitates, generated by metabolically active cells, were dissolved in methanol, and optical density was measured at 570 nm using a microplate reader. Half-maximal inhibitory concentration (IC_50_) values were estimated by fitting dose–response curves through non-linear regression analysis using GraphPad Prism software 10. Treatment selectivity was evaluated by calculating the selectivity index (SI), defined as the ratio between the IC_50_ obtained in fibroblast controls and that observed in melanoma cells (SI = IC_50__fibroblast/IC_50__melanoma), thereby indicating the relative cytotoxic preference toward tumor cells. For individual compound analyses, 2-AEH_2_P concentrations ranged from 1 to 100 mM, while metformin hydrochloride doses were selected based on preliminary response profiling and previously reported biological activity. In combination experiments, drug concentrations were normalized relative to the IC_50_ values determined for each agent alone in melanoma cells. Compounds were co-applied at varying molar proportions to generate a full dose–response interaction matrix, enabling subsequent assessment of pharmacological interaction patterns according to the Bliss independence reference model.

### 4.3. Evaluation of Cell Morphology and Selectivity Index

Melanoma cell and non-tumorigenic fibroblast controls were plated in 24-well culture plates and maintained for 24 h to ensure adequate cellular adhesion. After this stabilization period, cultures were subjected to a further 24 h exposure to increasing concentrations of 2-aminoethyl dihydrogen phosphate (2-AEH_2_P; 1–100 mM) and metformin hydrochloride (0.1–20 mM), administered either individually or in combined treatment regimens. Upon completion of the treatment interval, cellular morphology was inspected using an inverted light microscope (Nikon Eclipse TS100, Tokyo, Japan), and representative images were captured. Qualitative assessment focused on treatment-associated alterations, including modifications in cell shape, substrate detachment, cytoplasmic organization, membrane integrity, and characteristic apoptotic features. Observations were systematically compared with untreated control cultures to identify cytotoxicity-related phenotypic changes. To estimate treatment selectivity, the Selectivity Index (SI) was calculated according to the following relationship:

*Selectivity Index* = *IC_50_ fibroblasts*/*IC_50_ melanoma*

IC_50_ corresponds to the concentration required to reduce cellular viability by 50% relative to untreated controls. Elevated SI values were interpreted as indicative of preferential cytotoxic effects toward tumor cells. For single-agent conditions, both 2-AEH_2_P and metformin hydrochloride were evaluated independently across the specified concentration ranges. In combination experiments, metformin hydrochloride was applied at concentrations corresponding to its IC_50_ value, as well as at adjusted levels (−25% and +50% relative to IC_50_), together with graded doses of 2-AEH_2_P. This approach enabled characterization of interaction profiles and discrimination between additive and cooperative effects over a biologically relevant concentration window. Consistent with previously adopted criteria, SI values exceeding 2 were considered suggestive of tumor-selective activity [37].

### 4.4. Cell-Cycle Distribution and DNA Content Analysis

Melanoma cell lines (SK-MEL-28 and B16-F10) and fibroblast controls (FN1 and L929) were plated in 24-well culture plates at a density of 1 × 10^5^ cells per well and maintained for 24 h to ensure complete cellular attachment. After this equilibration period, cultures were exposed for an additional 24 h to increasing concentrations of 2-aminoethyl dihydrogen phosphate (2-AEH_2_P; 1–100 mM) and metformin hydrochloride (0.1–20 mM), administered either as single agents or in combined treatment regimens. At the end of the treatment interval, cells were harvested by enzymatic detachment using trypsin–EDTA solution, followed by two washes with phosphate-buffered saline (PBS, pH 7.4) to remove residual medium and serum components. Cell pellets were then fixed in cold 70% ethanol containing RNase A (DNase-free grade) to eliminate RNA interference during DNA quantification. Fixed samples were stored at −20 °C until cytometric analysis. Prior to data acquisition, cells were washed to remove ethanol and resuspended in staining solution composed of Triton X-100 and propidium iodide (PI). Samples were incubated for 30 min at room temperature in the absence of light to allow complete DNA intercalation. Cellular DNA content was subsequently measured using a FACSCanto II flow cytometer (BD Biosciences, San Jose, CA, USA), and the distribution of cells across distinct cell-cycle phases (G_0_/G_1_, S, and G_2_/M) was determined using ModFit LT software 5.0. To further characterize treatment-dependent modulation, selected cell lines were additionally exposed to concentrations corresponding to 50% of their previously determined IC_50_ values of 2-AEH_2_P, either alone or combined with metformin hydrochloride, under identical experimental conditions. For each sample, at least 10,000 gated events were collected and analyzed to ensure statistical reliability and accurate resolution of cell-cycle compartments.

### 4.5. Analysis of Cellular Proliferation by CFSE Labeling

Cellular proliferative behavior was examined using the fluorescent division-tracking dye carboxyfluorescein succinimidyl ester (CFSE-DA). Melanoma cell lines (SK-MEL-28 and B16-F10) and non-tumorigenic fibroblasts (FN1 and L929) were initially seeded in 24-well plates and maintained for 24 h to ensure adequate adhesion and stabilization. Following this period, cells were exposed to graded concentrations of 2-aminoethyl dihydrogen phosphate (2-AEH_2_P; 1–100 mM) and metformin hydrochloride (0.1–20 mM), administered either individually or in combination. After the treatment interval, cells were harvested by enzymatic dissociation using trypsin–EDTA solution, collected by centrifugation, and resuspended in phosphate-buffered saline. Samples were subsequently fixed in paraformaldehyde-containing buffer to preserve fluorescence signals and cellular integrity. CFSE fluorescence intensity, which decreases proportionally with successive cell divisions, was quantified using a FACSCanto II flow cytometer (BD Biosciences). Proliferation dynamics, including division profiles and proliferation index, were determined through computational analysis using ModFit LT software. This approach enabled comparative evaluation of treatment-induced effects on cell-cycle progression and replicative activity across experimental conditions.

### 4.6. Determination of Mitochondrial Membrane Potential (ΔΨm)

To assess mitochondrial functional status, melanoma cell lines (SK-MEL-28 and B16-F10) and fibroblast controls (FN1 and L929) were seeded in 6-well plates at a density of 1 × 10^5^ cells per well. After stabilization, cultures were exposed for 24 h to increasing concentrations of 2-aminoethyl dihydrogen phosphate (2-AEH_2_P) and metformin hydrochloride, administered either as single treatments or in combination. Following the treatment period, cells were harvested by gentle centrifugation and washed with phosphate-buffered saline (PBS, pH 7.4) to remove residual medium. Cell pellets were resuspended in FACS buffer consisting of PBS supplemented with bovine serum albumin to preserve cellular integrity during staining. Mitochondrial membrane potential (ΔΨm) was evaluated using the potentiometric fluorescent probe rhodamine 123, with samples incubated under controlled temperature and light-protected conditions to ensure stable dye accumulation. After staining, cells were washed to eliminate unbound dye and immediately subjected to flow-cytometric acquisition using a FACSCalibur cytometer (BD Biosciences). Fluorescence emission was collected in the FL1 channel following 488 nm excitation. For each sample, a minimum of 10,000 gated events was recorded to ensure statistical consistency. Mitochondrial polarization status was expressed as the mean fluorescence intensity (MFI) of rhodamine 123 relative to untreated controls. Reductions in fluorescence signal were interpreted as indicative of mitochondrial depolarization, consistent with early mitochondrial dysfunction.

### 4.7. Flow-Cytometric Evaluation of Intracellular Marker Expression

Melanoma cell lines (SK-MEL-28 and B16-F10) and fibroblast controls (FN1 and L929) were exposed for 24 h to 2-aminoethyl dihydrogen phosphate (2-AEH_2_P) and metformin hydrochloride under the experimental conditions previously described. Following treatment, cells were collected, washed with cold phosphate-buffered saline (PBS, pH 7.4), and fixed using paraformaldehyde solution to preserve cellular structures. To enable detection of intracellular antigens, membranes were permeabilized with Triton X-100 in PBS. Cells were subsequently incubated with primary monoclonal antibodies directed against proteins associated with mitochondrial regulation, apoptosis, and stress-response pathways, including cytochrome c, Bad, Bcl-2, p53, active caspase-3, and caspase-8. Antibody incubations were performed at low temperature with gentle agitation to ensure homogeneous labeling. After removal of unbound antibodies by washing in PBS supplemented with bovine serum albumin (BSA), samples were incubated with fluorochrome-conjugated secondary antibodies appropriate to the host species of each primary antibody. Secondary labeling was conducted under light-protected conditions to maintain fluorescence stability. Data acquisition was carried out on a FACSCanto II flow cytometer (BD Biosciences), with at least 10,000 gated events collected per sample to ensure statistical reliability. Fluorescence signals were analyzed using FlowJo™ v10 Software, and protein modulation was quantified based on variations in mean fluorescence intensity (MFI) measured within viable singlet populations, normalized to untreated controls. This strategy allowed sensitive detection of treatment-induced shifts in protein expression rather than reliance on absolute positivity thresholds. For combination experiments, sub-cytotoxic concentrations derived from previously determined IC_50_ values were employed to explore cooperative effects between 2-AEH_2_P and metformin hydrochloride. Mechanistic analyses were conducted using concentrations corresponding to 50% of each compound’s IC_50_ for the respective cell line, thereby minimizing confounding effects from extensive cell death while preserving biological responsiveness. Flow-cytometric gating procedures followed standard criteria, including exclusion of debris based on forward- and side-scatter parameters, discrimination of singlet events, and restriction of analysis to viable-cell populations prior to fluorescence quantification.

### 4.8. Drug Interaction and Synergy Assessment

The combined effects of 2-aminoethyl dihydrogen phosphate (2-AEH_2_P) and metformin hydrochloride were examined through a structured concentration–response design. Cells were treated for 24 h with independently varied concentrations of both compounds to characterize potential interaction patterns. Metformin hydrochloride was tested at three concentration levels defined relative to its previously determined IC_50_ value (−25%, IC_50_, and +50%), while 2-AEH_2_P was applied across a graded concentration range (1–100 mM). This experimental configuration produced a complete dose–response interaction matrix, enabling quantitative comparison between observed inhibitory effects and theoretical additive responses. Computational analysis of drug interactions was performed using the SynergyFinder platform, which evaluates deviations between experimentally measured responses and model-predicted outcomes. The Bliss independence reference model was adopted, as it assumes that both agents exert their biological effects through distinct and non-overlapping mechanisms. Interaction scores were interpreted according to established criteria: synergy scores exceeding +10 were classified as synergistic, values between −10 and +10 were considered indicative of additive behavior, and scores below −10 were interpreted as antagonistic interactions. This analytical framework allowed objective determination of whether combined treatment enhanced, preserved, or reduced cytotoxic responses relative to single-agent conditions.

### 4.9. Statistical Analysis

Quantitative data are presented as mean ± standard deviation (SD) derived from three independent experiments, each conducted with technical triplicates. Statistical analyses were performed using GraphPad Prism software. Comparisons among experimental conditions were carried out by one-way analysis of variance (ANOVA), followed by Tukey’s post hoc test for multiple comparisons. Prior to ANOVA application, normality and homogeneity of variance were assessed using the Shapiro–Wilk and Levene tests. Differences were considered statistically significant when *p* ≤ 0.05. Representative flow cytometry histograms illustrating population distribution and fluorescence profiles, acquired under standardized conditions with a consistent gating strategy across all samples in Supplementary Materials (Figures S1–S5).

## 5. Conclusions

This study demonstrated that 2-aminoethyl dihydrogen phosphate (2-AEH_2_P) induces significant antiproliferative and pro-apoptotic effects in melanoma cells through mitochondrial destabilization and activation of the intrinsic apoptotic pathway. Treatment promoted mitochondrial membrane depolarization, cytochrome c release, caspase activation, upregulation of pro-apoptotic regulators, and downregulation of Bcl-2. Metformin alone exerted moderate bioenergetic effects; however, its combination with 2-AEH_2_P markedly enhanced cytotoxicity, mitochondrial dysfunction, and apoptotic signaling. The association significantly reduced IC_50_ values and demonstrated synergistic interaction in melanoma models according to Bliss analysis. Although some cooperative responses were observed in fibroblasts, the magnitude of cytotoxicity remained lower than in melanoma cells, supporting a preferential tumor-directed effect. These findings indicate that metabolic co-targeting with metformin potentiates 2-AEH_2_P-induced mitochondrial apoptosis in melanoma, highlighting mitochondrial vulnerability as a promising target for combination-based drug repositioning strategies.

## Data Availability

The original contributions presented in this study are included in the article/Supplementary Material. Further inquiries can be directed to the corresponding author.

## Supplementary Materials

The following supporting information can be downloaded at https://www.mdpi.com/article/10.3390/ijms27125493/s1.
