# Supplementary material for: Metformin Enhances 2-Aminoethyl Dihydrogen Phosphate-Induced Mitochondrial Dysfunction and Apoptosis in Melanoma Cells
Source: Int J Mol Sci. 2026 Jun 18;27(12):5493. doi: 10.3390/ijms27125493 (PMC13299214; doi:10.3390/ijms27125493)
Supplement: Supplementary file 1 [file ijms-27-05493-s001.zip › ijms-4214840-supplementary.pdf]

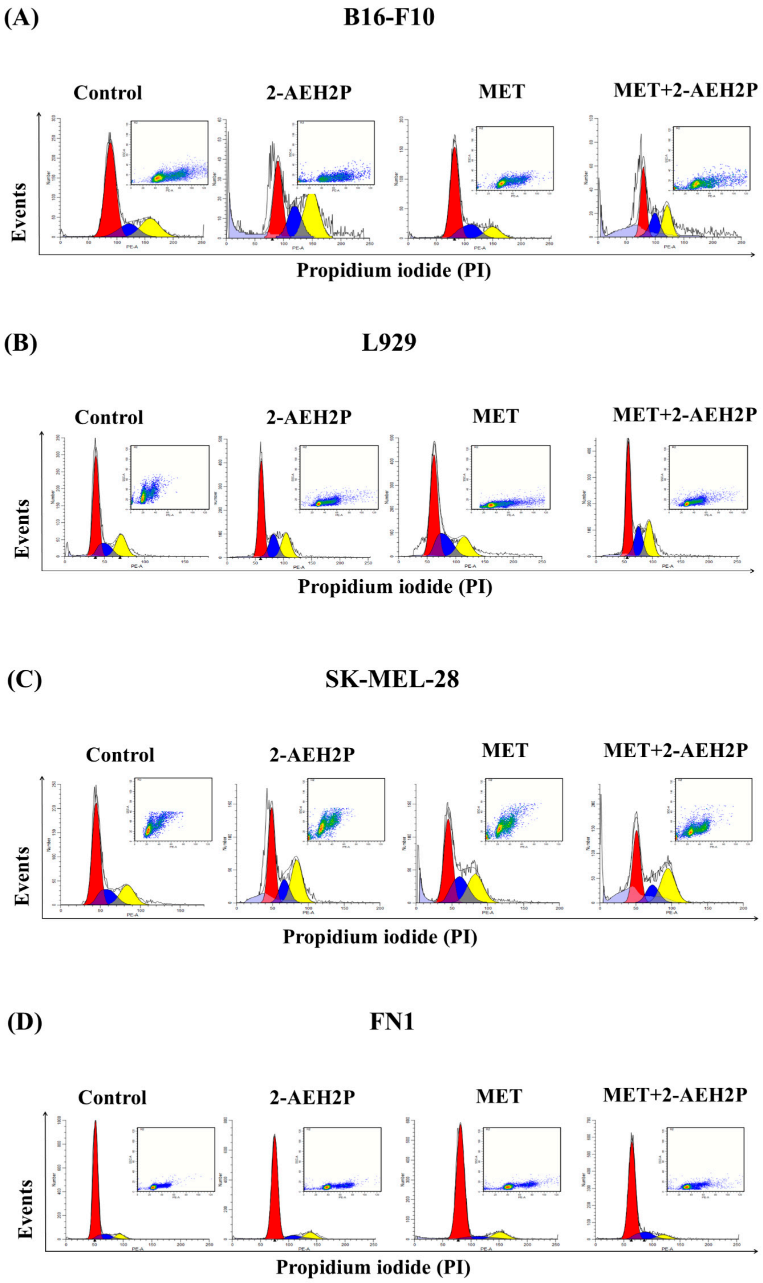

Supplementary Figure S1. Representative cell cycle distribution profiles analyzed by propidium iodide (PI) staining in melanoma and fibroblast cell lines. Representative histograms and dot plots of cell cycle distribution in **(A)** B16-F10, **(B)** L929, **(C)** SK-MEL-28, and **(D)** FN1 cells under control conditions, 2-AEH$_2$P treatment, metformin (MET) treatment, and combined treatment (MET + 2-AEH$_2$P). Data are presented as PI fluorescence intensity versus event count, with inset dot plots illustrating cell population distribution. The combined treatment results in increased accumulation of cells in S and G2/M phases, along with an increase in the sub-G1 population, indicative of cell cycle disruption and DNA fragmentation, particularly in melanoma cells.

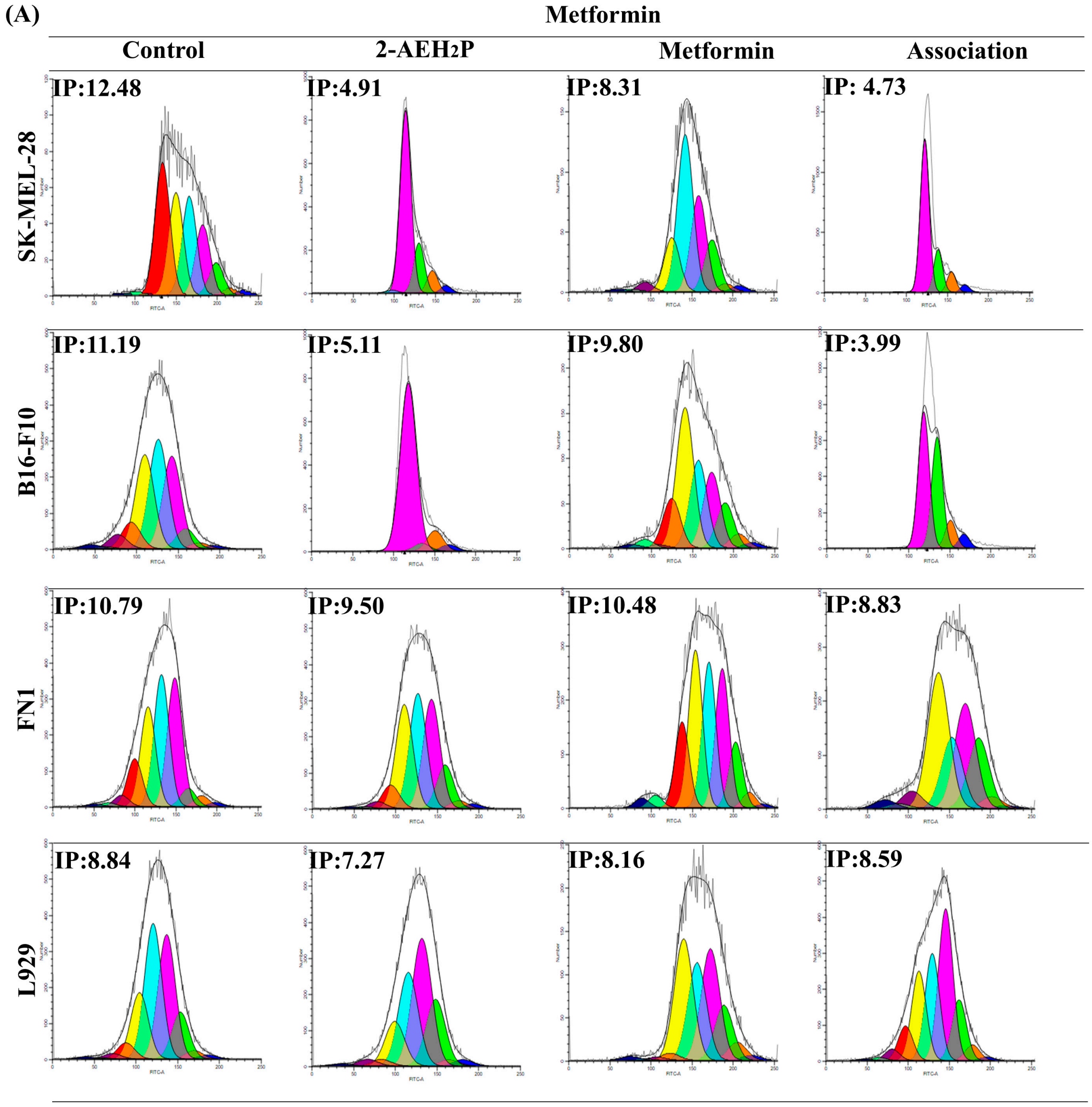

Supplementary Figure S2. Representative CFSE-based cell proliferation profiles following treatment with 2-AEH$_2$P, metformin, and their combination. Representative histograms of CFSE fluorescence intensity in SK-MEL-28, B16-F10, FN1, and L929 cells under control conditions, 2-AEH$_2$P treatment, metformin treatment, and combined treatment (metformin + 2-AEH$_2$P). Progressive dilution of CFSE signal reflects cell division, with leftward peak shifts indicating increased proliferation. The indicated IP values correspond to the proliferation index. Treatment with 2-AEH$_2$P and metformin, particularly in combination, results in reduced CFSE dilution in melanoma cells, consistent with inhibition of cell proliferation. In contrast, fibroblast cells display comparatively preserved proliferation profiles.

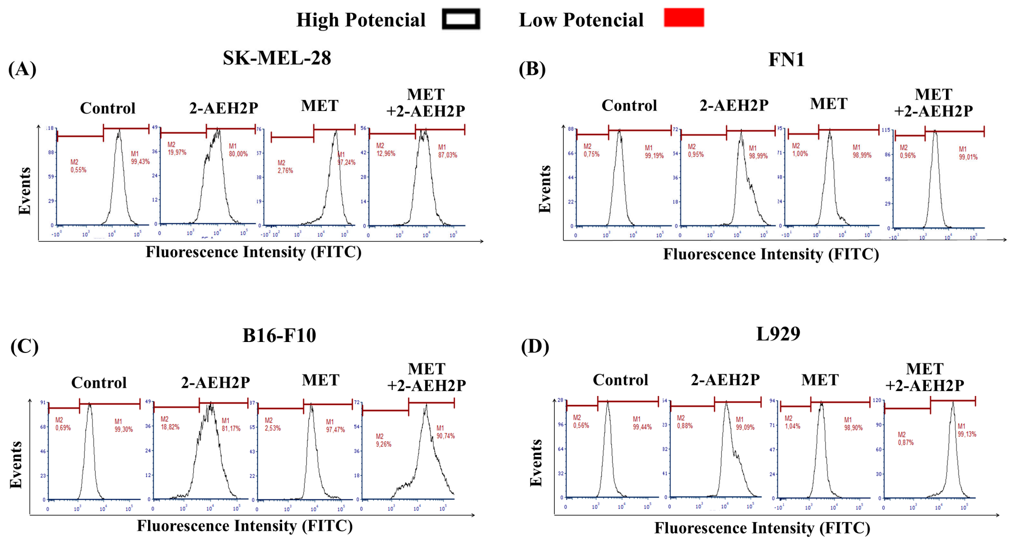

Supplementary Figure S3. Assessment of mitochondrial membrane potential following treatment with 2-AEH$_2$P, metformin, and their combination. Representative flow cytometry histograms showing mitochondrial membrane potential in **(A)** SK-MEL-28, **(B)** FN1, **(C)** B16-F10, and **(D)** L929 cells under control conditions, 2-AEH$_2$P treatment, metformin (MET) treatment, and combined treatment (MET + 2-AEH$_2$P). Fluorescence intensity (FITC) reflects mitochondrial potential status, with high mitochondrial membrane potential ($\Delta\Psi m$) indicated by intact polarization and low potential corresponding to mitochondrial depolarization. The combined treatment promotes a shift toward reduced mitochondrial membrane potential in melanoma cells, indicating enhanced mitochondrial dysfunction, while fibroblast cells exhibit comparatively preserved mitochondrial integrity.

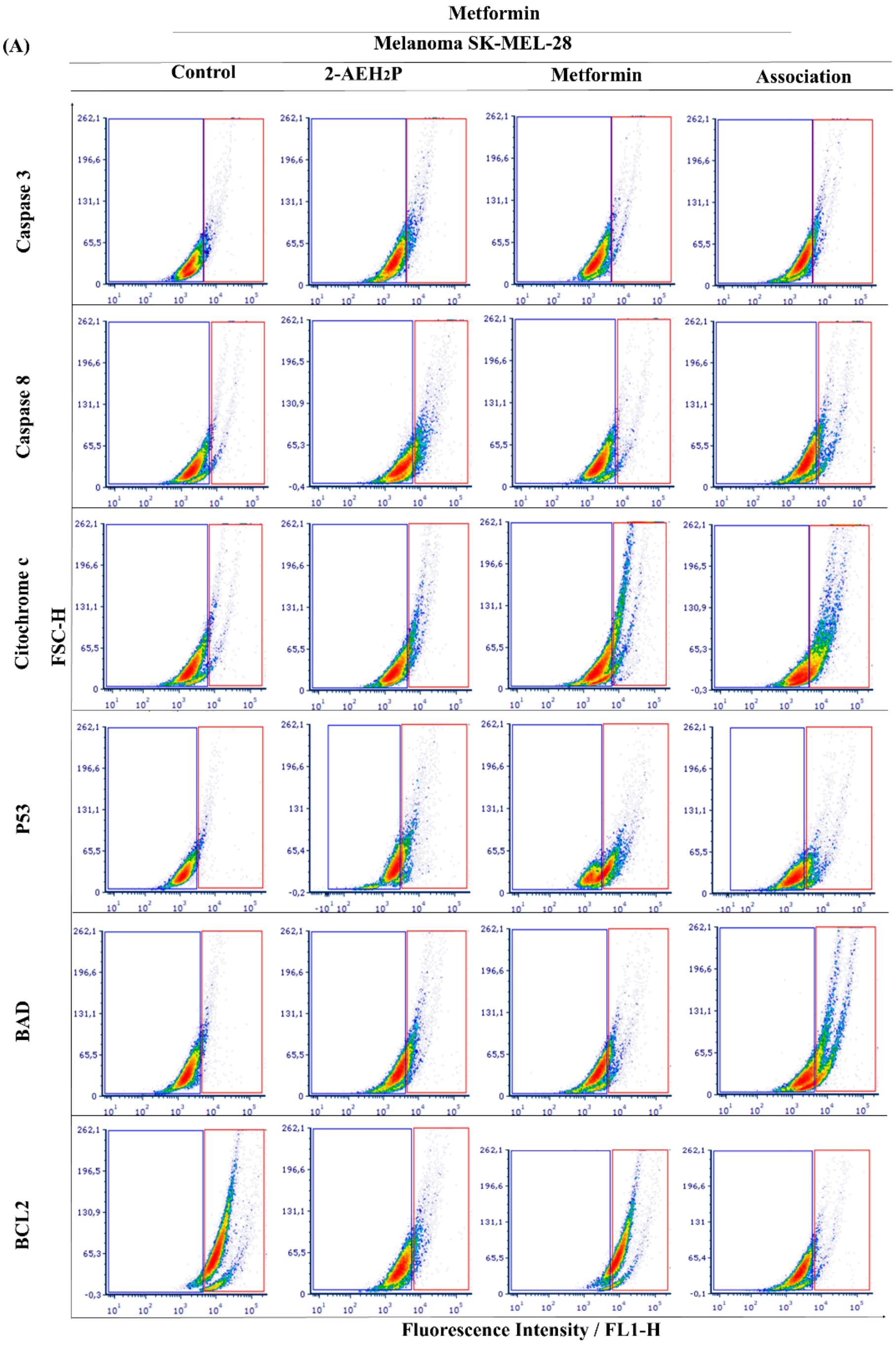

Supplementary Figure S4. Representative flow cytometry histograms of apoptosis- and mitochondrial-related markers in SK-MEL-28 melanoma cells. Representative dot plots illustrating the expression of caspase-3, caspase-8, cytochrome c, p53, BAD, and BCL-2 under control conditions, 2-AEH$_2$P treatment, metformin treatment, and combined treatment (metformin + 2-AEH$_2$P). Data are displayed as fluorescence intensity (FL1-H) versus forward scatter (FSC-H), with gated regions indicating positive cell populations. The combined treatment demonstrates enhanced activation of pro-apoptotic markers and modulation of mitochondrial-associated proteins compared to single treatments.

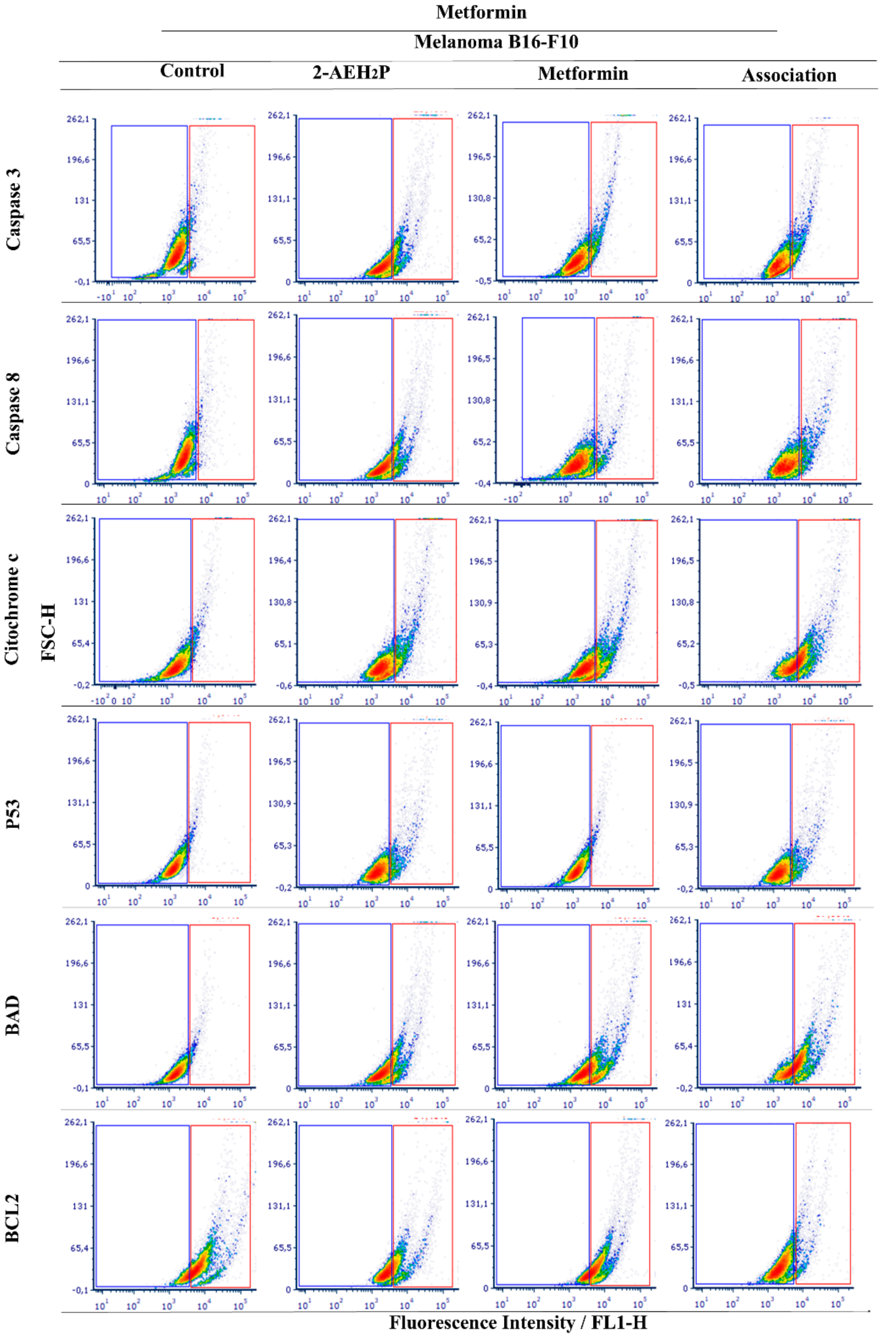

Supplementary Figure S5. Representative flow cytometry histograms of apoptosis- and mitochondrial-related markers in B16-F10 melanoma cells. Representative dot plots showing the expression of caspase-3, caspase-8, cytochrome c, p53, BAD, and BCL-2 in B16-F10 cells under control conditions, 2-AEH$_2$P treatment, metformin treatment, and combined treatment (metformin + 2-AEH$_2$P). Data are presented as fluorescence intensity (FL1-H) versus forward scatter (FSC-H), with gated regions indicating positive cell populations. The combined treatment promotes enhanced modulation of apoptotic and mitochondrial-associated markers compared to single treatments.
